# Upper respiratory virome signatures reflect COVID-19 severity and post-recovery shifts

**DOI:** 10.3389/fmicb.2026.1925299

**Published:** 2026-10-12

**Authors:** Mudita Gosain, Manjeet Kaur, Daizee Talukdar, Subhash Tanwar, Pradipta Jana, Sonali Porey Karmakar, Jyoti Verma, Susmita Bakshi, Kajal Kamboj, Priyanka Verma, Madhu Pareek, Sreevatsan Raghavan, Pallavi Kshetrapal, Shinjini Bhatnagar, Jayanta Bhattacharya, Pramod Kumar Garg, Nitya Wadhwa, Bhabatosh Das

**Affiliations:** 1 Maternal Child Health, BRIC-Translational Health Science and Technology Institute, NCR Biotech Science Cluster, Faridabad, India; 2 Microbial Research Centre, BRIC-Translational Health Science and Technology Institute, Faridabad, India; 3 Antibody Translational Research Program, Center for Virus Research, BRIC-Translational Health Science and Technology Institute, Faridabad, India; 4 Department of Gastroenterology, All India Institute of Medical Sciences, New Delhi, India

**Keywords:** bacteriophage, COVID-19, microbiome, SARS-CoV-2, virome

## Abstract

**Introduction:**

Severe acute respiratory syndrome coronavirus-2 (SARS-CoV-2) infection profoundly affects the upper respiratory tract, yet the dynamics of the resident virome across different stages of Coronavirus Disease 2019 (COVID-19) and recovery remain poorly understood.

**Methods:**

This study profiled alterations in the virome and microbiome in 143 SARS-CoV-2–positive adults, stratified by disease severity (Non-Severe, Severe, Critical), compared with 96 RT-PCR–negative controls. Nasal and oral swabs underwent targeted viral enrichment and next-generation sequencing, followed by virome profiling.

**Results:**

The respiratory virome of SARS-CoV-2-positive individuals was dominated by SARS-CoV-2, with co-detected viruses such as Human Mastadenovirus C, which increased with disease severity and persisting post-recovery. Alpha diversity was highest in Non-Severe cases and declined in Severe and Critical groups, suggesting increasing disruption of the virome with disease severity. Beta diversity showed clear separation between controls and cases, and across severity levels. LEfSe identified distinct viral signatures differentiating infection status, and logistic regression of key viral taxa accurately classified Critical versus post-recovery samples (AUC = 0.91). Although the study focused on viruses, bacterial taxa including Rothia mucilaginosa and Haemophilus parahaemolyticus were also detected with severity-linked variation, highlighting viral–bacterial interactions. Longitudinal paired analysis revealed consistent post-recovery enrichment of Streptococcus-specific bacteriophages across all severity groups, suggesting restoration of Streptococcus-associated phage dynamics during convalescence.

**Discussion:**

Overall, this study reveals SARS-CoV-2–associated alterations in the respiratory virome and microbiome and highlights specific microbial taxa with potential as biomarkers for disease severity and post-infection recovery.

## 1 Introduction

Coronavirus Disease 2019 (COVID-19), caused by *Severe Acute Respiratory Syndrome Coronavirus 2* (SARS-CoV-2), was declared a global pandemic by the World Health Organization (WHO) on March 11, 2020 (World Health Organization, 2020). The outbreak originated as a cluster of pneumonia cases in Wuhan, China, and rapidly escalated into a global health crisis. SARS-CoV-2 and its emerging variants have been responsible for widespread morbidity and mortality, with many affected individuals. The substantial mortality associated with COVID-19, together with marked heterogeneity in disease severity, underscores the need to identify virome signatures associated with clinical outcomes and post-recovery changes. The gold standard for COVID-19 diagnosis is the detection of viral RNA in upper respiratory tract (URT) specimens using reverse transcription polymerase chain reaction (RT-PCR) (Wölfel et al., 2020). However, RT-PCR has an estimated false negative rate of at least 20%, which may result from various factors such as sampling errors, low viral load, or genomic mutations affecting primer binding sites (Kucirka et al., 2020).

Microbial communities colonize all environmentally exposed surfaces of the human body, including the mucosal lining of the respiratory tract. Over the course of evolution, these microorganisms including the virome, bacteriome, and mycobiome have co-evolved with the human host, many establishing themselves as commensals without causing disease. These resident microbial communities play a pivotal role in maintaining respiratory health by modulating immune responses and contributing to mucosal homeostasis (Honda and Littman, 2016). The respiratory microbiome begins to establish at birth and stabilizes during early childhood, forming a complex and dynamic ecosystem. One of the essential functions of this indigenous microbiota is to provide colonization resistance, whereby resident microbes inhibit the proliferation and invasion of pathogenic organisms. This defense mechanism not only limits pathogen colonization but also contributes to the structural and immunological maturation of the respiratory system (Man et al., 2017).

Upper respiratory tract (URT) is the primary interface between the host and airborne microorganisms, including SARS-CoV-2, the causative agent of COVID-19. Given this critical role, it is plausible that SARS-CoV-2 infection perturbs the URT microbiota (Zhu et al., 2020). However, the extent and nature of alterations in the non-SARS-CoV-2 virome during and after infection remain poorly understood. SARS-CoV-2 infects epithelial cells of the URT through binding of its spike (S) protein to the angiotensin-converting enzyme 2 (ACE2) receptor, with the host transmembrane serine protease 2 (TMPRSS2) facilitating viral entry (Hoffmann et al., 2020). These interactions can trigger local inflammation, disrupt epithelial barriers, and potentially alter the composition and structure of the resident microbial communities. While most research has focused on SARS-CoV-2 itself, relatively little is known about how this infection perturbs the broader community of resident viruses, distinct from SARS-CoV-2 as well as commensal and pathogenic bacteria inhabiting the URT.

### 1.1 What is known so far

Microbiome and Virome Profiling of the URT in SARS-CoV-2 Infection: Indian and Global Perspectives. Recently, research on the URT microbiome in respiratory infection with COVID-19 has been the focus globally, yet there is limited information from the Indian or Asian population on the characterization of the virome. To date, there is a paucity of information available in peer-reviewed literature on the characterisation of the upper respiratory tract virome in COVID-19 and the global data on virome richness and abundance and its temporal dynamics remains sparce. Thus, national data on virome diversity, richness, or temporal dynamics in this context remain entirely lacking. There are a few groups which has worked on bacterial microbiota using 16S rRNA sequencing. A large cross-sectional study from Council of Scientific and Industrial Research -Centre for Cellular and Molecular Biology (CSIR-CCMB) profiled 589 individuals across three pandemic waves, revealing wave-specific dysbiosis, including consistent depletion of Rickettsiaceae and enrichment of *Staphylococcus*, *Thermus*, and *Aerococcus* in symptomatic cases (Bose et al., 2023).

Kumar et al. (2022) investigated on deceased patients, recovered individuals and healthy controls. The group reported that deceased patients exhibited higher species richness and enrichment of pathogenic genera like *Corynebacterium* and *Serratia*, while recovered individuals showed increased abundance of *Burkholderia* and *Ochrobactrum*. The Microbiota of healthy controls were dominated by *Pseudomonas* and *Veillonella*, suggesting a potential protective role of commensals (Kumar et al., 2022). Studies from other countries have provided broader insights into the respiratory virome and microbiome during COVID-19. For instance, Merenstein et al. (2021) investigated the respiratory tract microbiome in 83 hospitalized COVID-19 patients and compared their profiles with those of non-COVID-19 patients and healthy controls. Building on these observations, it is hypothesized that SARS-CoV-2 infection may alter the composition and diversity of both the virome and bacterial microbiome of the upper respiratory tract.

## 2 Materials and methods

To investigate this, nasopharyngeal and oropharyngeal samples were collected and retrieved for targeted viral enrichment and next-generation sequencing. These samples were stored in Viral Transport Media (VTM), from individuals with active COVID-19 and were compared with those from SARS-CoV-2 negative individuals (controls) without recent respiratory illness. The samples from both sites were included but not analysed separately.

To further understand the temporal dynamics of these microbial shifts, a longitudinal, paired-sample design was implemented where samples were collected from the same individuals during the active phase of infection and again post-recovery. This approach enabled direct within-host comparison, minimizing inter-individual variability and allowing us to assess how the non-SARS-CoV-2 microbial communities evolve during infection and convalescence. No longitudinal follow-up samples were collected from the control group; follow-up sampling was limited to SARS-CoV-2 positive individuals’ assess within-host virome temporal dynamics and to observe the restoration of dysbiosis seen during acute infection period.

### 2.1 Sample size

To detect an anticipated difference of microbiome in at least 25% (i.e., 40% abundance in cases), greater abundance compared to the average abundance in controls (15% abundance), and assuming 5% Type I error rate with 90% power, a total of 170 participant are required, with 85 in each arm.

### 2.2 Study design and participants’ information

The study was conducted between 2020 and 2024 at the COVID-19 testing centres operated by Translational Health Science and Technology Institute (THSTI) and the Employees’ State Insurance Corporation Medical Hospital (ESICMH), Faridabad. The target population included individuals presenting for RT-PCR testing for SARS-CoV-2 at these centres. A case–control study design was leveraged to profile and compare the composition and diversity of the upper respiratory tract virome between individuals with active SARS-CoV-2 infection and those without evidence of active respiratory or gastrointestinal infection, with a focus on characterizing dysbiosis associated with SARS-CoV-2. Cases were defined as individuals who tested RT-PCR positive for SARS-CoV-2. Controls were defined as individuals who tested RT-PCR negative (n = 96) at the time of sample collection, reported no active respiratory symptoms, had not taken antibiotics in the preceding 6 months, and had no history of SARS-CoV-2 infection or known exposure in the preceding 6 months. All participants provided written informed consent prior to enrolment. Demographic and clinical data including age, sex, symptoms, comorbidities, vaccination status, and healthcare utilization (for hospitalized participants) were obtained through a structured interview. Participants were approached for post-recovery follow-up, during which repeat URT samples were collected at least 6 months after their initial enrolment.

### 2.3 Ethics permission

All necessary ethical approvals were obtained from relevant committees at participating institutions. Written informed consent was obtained from all participants prior to enrolment. The ethics permission was obtained from the institutional ethics committee, THS 1.8.1/(156) dated 07 December 2023.

### 2.4 Sample collection, packaging and transport

Nasopharyngeal and oropharyngeal swabs were collected from suspected COVID-19 patients using Dacron, polyester flocked, or cotton swabs in 3 mL of HiMedia’s HiViral™ Viral Transport Media (VTM) in accordance with ICMR and WHO guidelines. Trained health care workers performed the collection using appropriate personal protective equipment. Post-collection, samples were barcoded, packaged by trained personnel, and transported under cold-chain conditions (4 °C) using ice or gel packs. A dedicated transport team ensured timely delivery to the testing laboratories, with continuous monitoring of the temperature.

### 2.5 Viral RNA isolation, quantification, quality assessment and storage

Clinical specimens were transported to the Bioassay Facility at THSTI following standardized biospecimen transport and storage protocols. Viral RNA was extracted from the VTM using the QIAamp Viral RNA Mini Kit (QIAGEN) (Cat no. 52904), according to the manufacturer’s instructions. The extraction protocol involved sample lysis, followed by binding of viral nucleic acids to the silica membrane in the presence of a binding buffer and isopropanol. All the residual samples were finally stored at Biorepository facility at THSTI.

### 2.6 Library preparation and sequencing using respiratory virus oligo panel (RVOP) and Illumina MiSeq sequencing platform

Complementary DNA (cDNA) was synthesised from the extracted RNA to target RNA viruses, followed by tagmentation and indexing using Illumina unique dual (UD) indexes. The libraries were purified using AMPure XP beads (Beckman Coulter). Viral sequence enrichment was performed through multiplex PCR using the Illumina Respiratory Virus Oligo Panel (RVOP), which selectively amplifies genomic regions from a broad range of respiratory viruses. RVOP is a proprietary enrichment panel developed and manufactured by Illumina, Inc. (San Diego, California, USA) as part of their Respiratory Virus Enrichment Kit for Illumina sequencing workflows.

The enriched libraries were normalised, pooled, and prepared for sequencing. The pooled libraries were then denatured with NaOH and diluted to a final concentration of 12 pM according to the Illumina protocol. Sequencing was performed on the Illumina MiSeq platform using paired-end 75-cycle chemistry (2 × 75 bp), generating high-quality viral sequence data.

### 2.7 Bioinformatics analysis

Quality control of raw Fastq files was initially performed using FastQC. Low-quality reads and adapter sequences were removed using Fastp version 0.20.1, ensuring high-confidence downstream analysis. Filtered reads were then aligned to the human reference genome (GRCh38) using Bowtie2 version 2.5.2 in end-to-end mode to remove potential host-derived sequences. Taxonomic profiling of non-host reads was conducted using Kraken2 (Wood and Salzberg, 2014), employing the RefSeq standard database (release 220), in which low-complexity regions were masked to reduce classification artefacts. This step enabled the comprehensive detection of both viral and bacterial taxa from the sequencing data.

Read counts assigned to viral and bacterial taxa were parsed and filtered using a custom Bash script to retain only confidently classified taxa. The processed abundance tables were subsequently merged and formatted using an in-house R script for downstream statistical analysis.

### 2.8 Statistical methods

Analysis was performed using the R software package. Alpha diversity was assessed using the estimate richness function from the phyloseq package. Statistical significance across more than two groups was evaluated using the non-parametric Kruskal-Wallis test, followed by Dunn’s post-hoc test. Beta diversity was assessed using Bray–Curtis dissimilarity, and ordination was performed via Principal Coordinates Analysis (PCoA) using the vegan package. Differences in beta diversity between groups were tested using PERMANOVA (Permutational Multivariate Analysis of Variance) implemented through the adonis function from vegan. Bacterial and viral read counts were normalized to relative abundance (%) at the individual-sample level using ampvis2. Taxa were aggregated at the species level. The mean relative abundance of each bacterial species was calculated across individual samples within each clinical condition group and visualized using the amp_heatmap function. Only the top taxa were displayed, with a minimum abundance threshold of 0.1%. The colour scale was displayed on a log10 scale.

Differentially abundant taxa were identified using the *run_lefse* function in the microbiomeMarker package. To evaluate whether viral features could discriminate disease status, logistic regression models were generated using the *run_sl* function. Model performance was assessed by plotting receiver operating characteristic (ROC) curves with the *plot_sl_roc* function.

## 3 Results

### 3.1 Clinical characteristics of the study population

143 individuals were enrolled across the outpatient department (OPD = 59 participants), inpatient ward (IPD = 77 participants) and Intensive Care Units (ICU = 7 participants). Individuals who tested positive for COVID-19 by RT-PCR were enrolled and categorized into Critical, Severe, and Non-severe groups according to the revised WHO Global Clinical Platform for COVID-19 disease severity definitions (World Health Organization, 2021). The operational criteria used for each category are summarized in Supplementary Table S1. Non-severe: 113 participants (79.0%), Severe: 17 participants (11.9%), and Critical: 13 participants (9.1%). The cohort comprised 79 males (55.2%) and 64 females (44.8%), with a mean age of 42.3 years. These participants samples were also sequenced to identify their variants. There were 02 cases of Alpha, 71 cases infected with Delta (B.1.617.2), 02 cases with AY sub-lineages (AY.4.2, AY.114), 03 cases with Kappa variant and 65 cases with variant unassigned or lineage not detected.

All participants were approached for post-recovery follow-up, and of them, 41 consented to provide follow-up samples, collected at least 06 months after their initial enrollment. Reasons for non-collection among the remaining participants included: declined to provide samples (*n* = 13), out-of-station/relocated to an out-of-study area (n = 48), no response/uncontactable (*n* = 30), and death (*n* = 11). These follow-up samples included individuals from all three severity groups. The recovered samples were analyzed collectively to assess longitudinal changes. For the control group, individuals who were RT-PCR-negative (n = 96) at the time of sample collection with no active respiratory symptoms, had not taken antibiotics in the last 6 months, had no history of SARS-CoV-2 infection in the last 6 months, or had recent exposure were recruited. (Table 1). The study flow and classification of participants for this study are shown in Supplementary Figures S1a,b.

**Table 1:** Cohort profile.

Total participants (*n* = 239)			
Characteristics		Positive (n = 143)	Negative (*n* = 96)
Age group	<18	10 (6.99%)	7 (7.29%)
	18–59	105 (73.42%)	82 (85.41%)
	≥60	27 (18.88%)	7 (7.29%)
Gender	Male	79 (55.24%)	43 (44.79%)
	Female	64 (44.75%)	53 (55.20%)
Frontline worker	Yes	12 (8.39%)	1 (1.04%)
	No	131 (91.60%)	95 (98.95%)
Place of enrolment	ICU	7 (4.89%)	NA
	Ward	46 (32.16%)	NA
	OPD	90 (62.93%)	96 (100%)
Disease Severity	Critical	13 (9.1%)	NA
	Non-severe	113 (79.0%)	NA
	Severe	17 (11.90%)	NA
Vaccine status* (as on date of sample collection)	Yes	0	71 (73.95%)
	No	0	6 (6.25%)
Health care use (acute disease)			
ICU	Yes	12 (08.39%)	NA
	No	131 (91.60%)	NA
Oxygen	Yes	20 (13.98%)	NA
	No	123 (86.01%)	NA
Ventilator	Yes	04 (02.79%)	NA
	No	139 (97.20%)	NA
Death (due to COVID-19)	Yes	11 (07.69%)	NA
	No	132 (92.30%)	NA

*Completely vaccinated individuals are defined as individuals who have received two doses of vaccine and have passed 28 days from the date of dose 2.

Of the 143 sequenced participants, comorbidity data like metabolic disorders, chronic infections were available for a subset of 73 individuals, as clinical information was obtained only from those who completed comprehensive intake interviews or had complete medical records at the time of enrollment.

A total of 73,661,139 reads were obtained after sequencing, of which 71,878,224 reads (97.58%) were classified by Kraken2. Among the classified reads, 89.58% were of viral origin, consistent with the expected enrichment of viral particles in the sample. Bacterial reads accounted for 10.39%, which may represent residual bacterial contamination or prophage sequences integrated into bacterial genomes. A very small proportion (0.03%) of reads were assigned to taxa other than bacteria and viruses namely eukaryotes and archaea likely reflected low levels of host or environmental DNA in the samples. To specifically investigate co-infecting viral species, reads corresponding to SARS-CoV-2 were removed from the dataset. The objective of the study was to elucidate the presence of additional viral taxa beyond SARS-CoV-2 using a target enrichment approach based on hybridization capture. As expected, the respiratory virome was overwhelmingly dominated by SARS-CoV-2. However, after removal of SARS-CoV-2 reads, limited co-detection of other respiratory viruses was observed, indicating a low abundance and/or absence of common viral co-infections in the majority of samples.

### 3.2 Alteration in virome diversity of the upper respiratory tract reflects COVID-19 severity and recovery trajectory

To assess whether virome diversity was associated with COVID-19 severity, alpha diversity was measured between RT-PCR-positive (cases) and RT-PCR-negative samples (controls). Alpha diversity was significantly elevated in RT-PCR-positive samples compared to RT-PCR-negative ones across all indices (Figure 1). RT-PCR positive individuals exhibited significantly greater richness as compared to RT-PCR negative ones, as indicated by Observed (*p* < 0.0001) and Chao1 (*p* < 0.001) indices (Figure 1a,b). Furthermore, Shannon and Simpson diversity indices were also significantly higher in the RT-PCR positive group (*p* < 0.0001), suggesting not only a greater number of taxa but also a more even distribution within the viral community (Figures 1c,d). Principal Coordinates Analysis (PCoA) based on Bray–Curtis dissimilarity revealed a clear separation between RT-PCR Negative and RT-PCR Positive samples (Figure 1e). The two groups clustered distinctly, with minimal overlap, indicating significant differences (PERMANOVA, *R*^2^ = 0.192, *p* = 0.001) in viral community composition.

**Figure 1:**
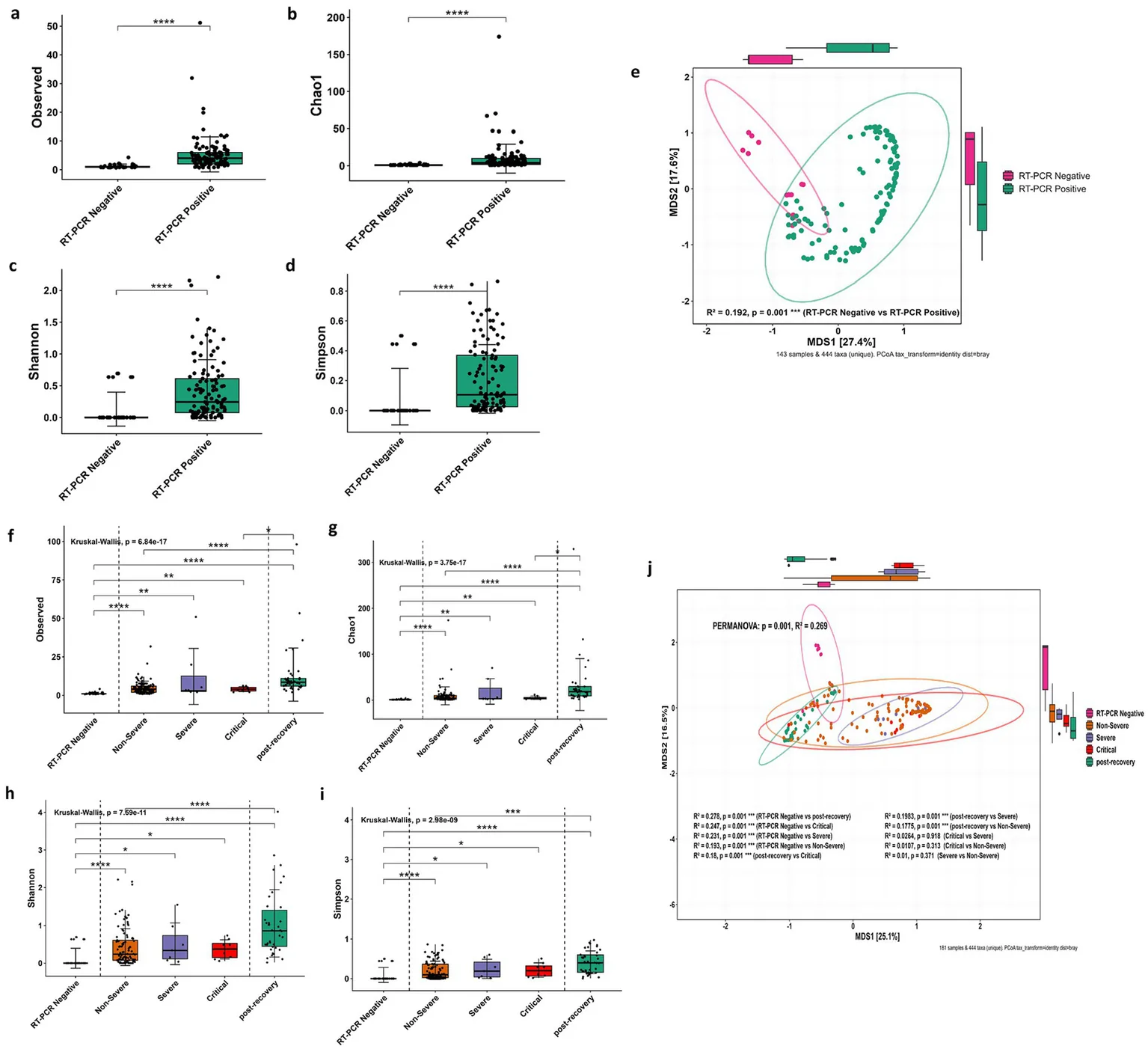
Alpha and beta diversity analyses across RT-PCR status and clinical severity groups: alpha diversity analysis comparing RT-PCR Positive and RT-PCR Negative groups **(a–d)**: Observed **(a)**, Chao1 **(b)**, Shannon diversity index **(c)**, and Simpson diversity index **(d)** indicate significantly higher alpha diversity in RT-PCR positive individuals than in RT-PCR negative controls across most metrics. Beta diversity analysis Bray–Curtis distance-based Principal Coordinate Analysis (PCoA) shows clear separation between RT-PCR positive and negative groups **(e)**. Alpha diversity across clinical severity groups (RT-PCR Negative, Non-Severe, Severe, Critical, and Post-Recovery) **(f–i)**: Boxplots show observed richness **(f)**, Chao1 **(g)**, Shannon **(h)**, and Simpson **(i)** indices. A significant reduction in diversity is observed in the Severe and Critical groups compared to Non-Severe and Post-Recovery samples. The post-recovery group showed partial restoration of diversity. Statistical significance between groups was tested using Kruskal–Wallis test followed by the Dunn test. Beta diversity (Bray–Curtis PCoA) stratified by disease severity and recovery status reveals distinct clustering by group **(j)**. Statistical significance between groups was tested using PERMANOVA, with *p*-values. *p < 0.05, **p < 0.01, ***p < 0.001.

Furthermore, RT-PCR positive samples were stratified by clinical severity (Non-severe, severe, critical). All three severity groups showed a statistically significant (*p* < 0.05) increase in diversity compared to RT-PCR negative group. Interestingly, the post-recovery group demonstrated the highest richness and evenness among all the groups (Figure 1f–i). Beta diversity analysis using PCoA (Bray-Curtis) revealed distinct clustering patterns across clinical groups (Figure 1j). RT-PCR Negative samples formed a well separated cluster, while RT-PCR Positive subgroups showed varying degrees of overlap and dispersion. PERMANOVA confirmed significant differences (*R*^2^ = 0.269, *p* = 0.01) in community composition between RT-PCR negative samples and each of the RT-PCR positive subgroups: Post-Recovery (*R*^2^ = 0.278, *p* = 0.001); Critical (*R*^2^ = 0.247, *p* = 0.001); Severe (*R*^2^ = 0.231, *p* = 0.001); Non-Severe (*R*^2^ = 0.193, *p* = 0.001). Furthermore, significant compositional differences were also noted between RT-PCR Positive subgroups and post-recovery group: Post-recovery vs. Severe (*R*^2^ = 0.1983, *p* = 0.001), post-recovery vs. Non-Severe (*R*^2^ = 0.1775, *p* = 0.001), indicating significant differences in virome composition.

### 3.3 Compositional variation of virome among groups

The Venn diagram highlights unique and overlapping viral taxa detected in different clinical stages (Figure 2a). Notably, Streptococcus phage PH10 was enriched specifically in post-recovery samples, Human Mastadenovirus C was common between active COVID-19 conditions (Non-Severe, Severe, and Critical) and Post-Recovery group. In addition, Bat coronavirus BM48-31BGR/2008 was common between Critical and Severe groups, indicating differential phage dynamics in disease vs. recovery.

**Figure 2:**
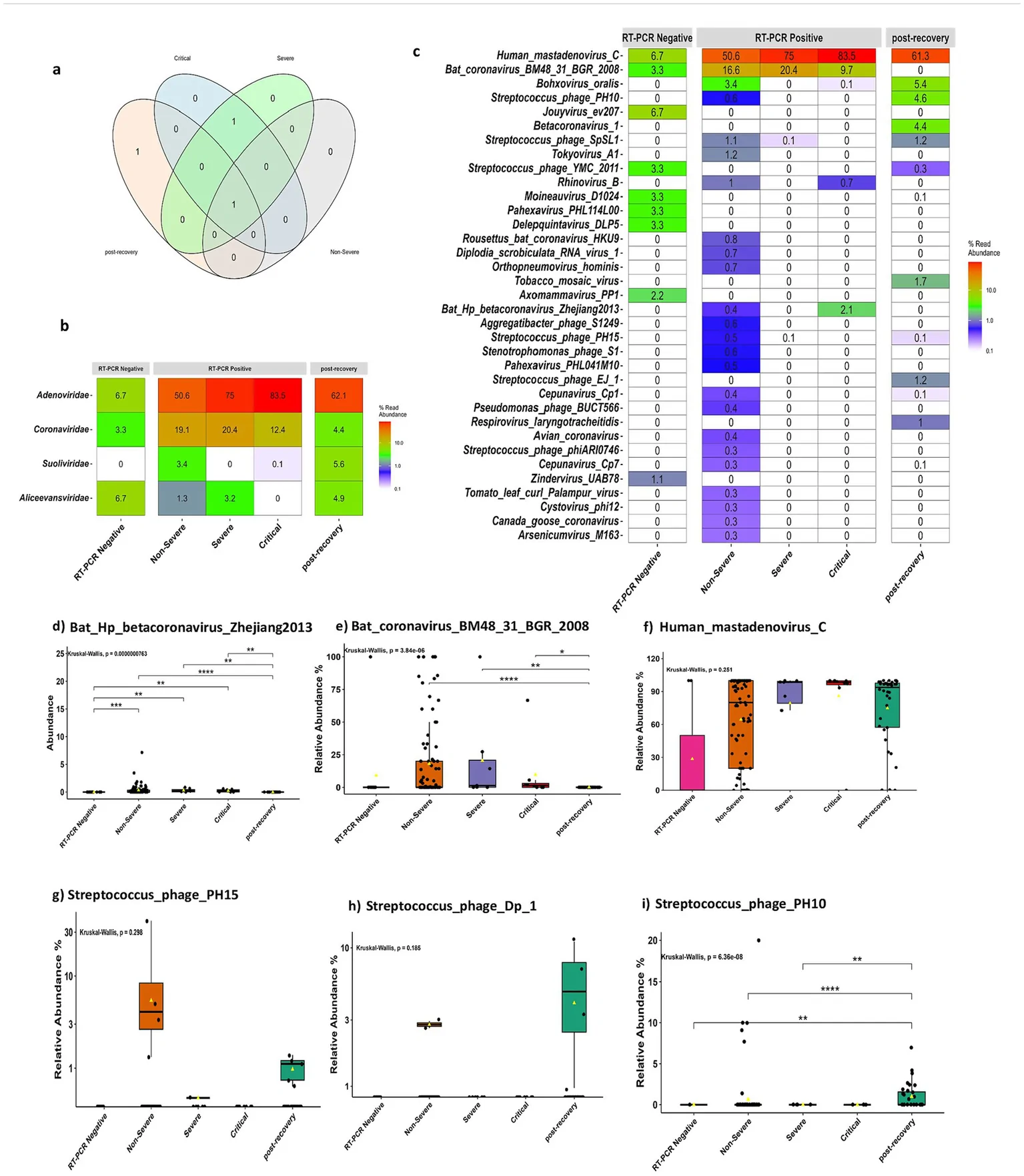
Composition and abundance of upper respiratory tract virome across COVID-19 severity groups and recovery phase: Venn diagram represents the prevalence at 50% of core viruses across different clinical groups **(a)**. Heatmap showing the distribution of viral taxa across different clinical categories: RT-PCR Negative, Non-Severe, Severe, Critical, and Post-Recovery. At family **(b)** and species level. **(c)** Bar plots showing the abundance of selected viral species of interest across five clinical groups **(d–i)**: Bat_Hp_betacoronavirus_Zhejinang2013, Bat_coronavirus_BM48_31_BGR_2008, Human Mastadenovirus C, Streptococcus phage PH15, Streptococcus phage Dp 1, Streptococcus phage PH10. Statistical comparisons between groups were performed using Kruskal–Wallis test. *p < 0.05, **p < 0.01, ***p < 0.001.

The virome composition at the family level is shown in Figure 2b, with adenoviridae was the most prevalent family, followed by Coronaviridae and Suoliviridae. A marked increase in Adenoviridae was observed with increasing COVID-19 severity as compared to RT-PCR negative, rising from just 6.7% in RT-PCR negative individuals to 50.6%, 75%, and 83.5% in the Non-Severe, Severe, and Critical groups, respectively. This high prevalence persisted even in the post-recovery group (62.1%), suggesting sustained upper respiratory colonization or reactivation. Interestingly, members of the Suoliviridae were nearly undetectable in RT-PCR negative, severe, and critical cases but showed modest enrichment in the non-severe (3.4%) and post-recovery (5.6%) groups, indicating a possible re-establishment of low-abundance resident viruses during recovery.

Overall, 446 viral species belonging to 59 different families were detected. Bat coronavirus BM48-31 BGR 2008 were notably abundant in RT-PCR positive groups, as compared to RT-PCR negative samples. Human Mastadenovirus C emerged as the most abundant viral taxon in RT-PCR positive individuals, showing a dramatic increase compared to RT-PCR negative samples (50.6%–83.5% vs. 6.7%), respectively. Its relative abundance increased progressively with disease severity, peaking in the Critical group (83.5%), and remained elevated even in the post-recovery group (61.3%). This suggests a strong association between Human Mastadenovirus C and SARS-CoV-2 co-infection or immune modulation, potentially contributing to disease progression or delayed recovery. Several Streptococcus phages (Streptococcus phage YMC-2011, Streptococcus phage EJ-1) were detected only in RT-PCR negative or post-recovery groups at low abundance. Other commensal or environmental viruses like Pahexavirus PHL114L00, Moineavirus D1024, Jouyvirus ev207, Zindervirus UAB78 and Delepquintavirus DLP5 were exclusively present in RT-PCR negative group (Figure 2c).

Specific bar plot shows that Human Mastadenovirus C (Figure 2F) was significantly enriched in RT-PCR positive samples and decreased post-recovery. Several Streptococcus phages were detected such as Streptococcus phage PH15 (Figure 2g) Streptococcus phage Dp1 (Figure 2h), Streptococcus phage PH10 (Figure 2i), and others showed a significant rise in post-recovery samples compared to all disease states.

### 3.4 Distinct virome signatures differentiate SARS-CoV-2 infection status and disease severity

To identify virome features associated with SARS-CoV-2 infection, we performed differential analysis using Lefse comparing RT-PCR positive and RT-PCR negative individuals. LEfSe scores are shown in Supplementary Table S2. This revealed that Human Mastadenovirus *C* was significantly enriched in RT-PCR positive subjects, whereas Moineauvirus D1024 (Streptococcus phage D1024), Pahexavirus PHL114L00 (Propionibacterium phage), and Jouyvirus ev207 (Escherichia phage), Zindervirus UAB78 (Escherichia phage) were more abundant in RT-PCR negative individuals (Figure 3a). These findings suggest that SARS-CoV-2 infection is associated with specific alterations in the virome composition.

**Figure 3:**
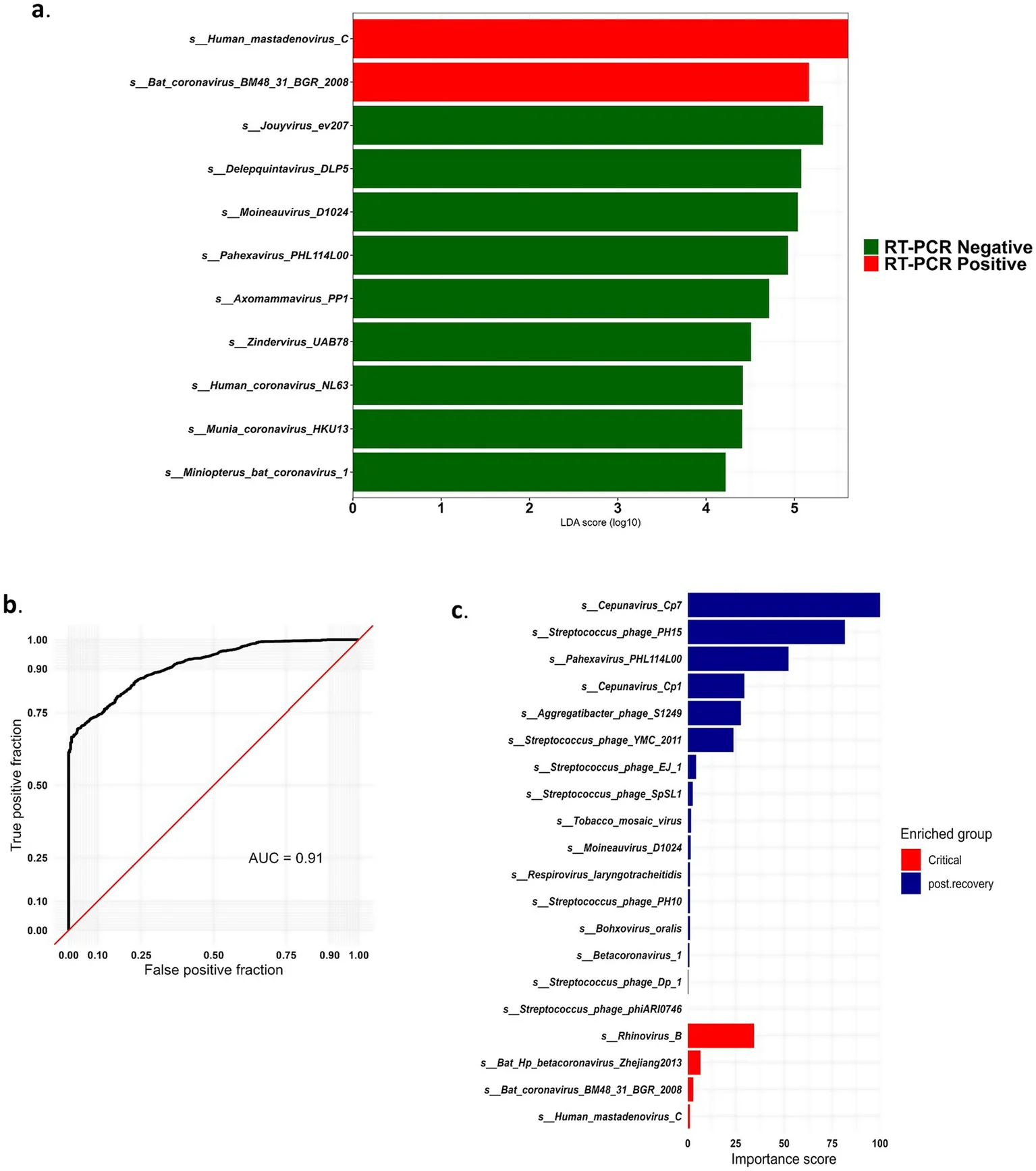
Differential abundance analysis and classification of viral taxa across COVID-19 clinical groups. **(a)** Linear discriminant analysis (LDA) Effect Size (LEfSe) plot showing viral taxa differentially enriched between RT-PCR Negative and RT-PCR Positive groups. **(b)** Receiver Operating Characteristic (ROC) curve evaluating the discriminatory performance of a Random Forest classification model based on top20 viral signatures between critical and post-recovery groups. The model showed high classification accuracy with an Area under the Curve (AUC) of 0.91. **(c)** Random Forest feature importance plot showing the top viral species contributing to classification among critical and post-recovery groups.

Due to the limited sample size in the critical group (*n* = 13), conventional statistical approaches such as differential abundance testing with robust correction for multiple comparisons were not feasible without compromising statistical power. To overcome this limitation while still capturing meaningful virome dynamics associated with disease severity, we employed logistic regression modeling, a method well suited for small sample sizes when appropriately regularized. To further explore virome dynamics related to disease progression, we focused exclusively on RT-PCR positive individuals, specifically the critical phase representing peak illness to those in the post-recovery phase, reflecting clinical resolution and potential normalization of host–virus interactions. This comparison enabled the identification of viral and phage species that are differentially enriched during acute disease versus recovery, offering insight into virome contributions to disease severity and resolution. To assess the predictive value of these viral species, we applied logistic regression modeling using the top 20 viral taxa. The model achieved an area under the curve (AUC) of 0.91, indicating excellent classification performance between Critical and Post-Recovery individuals (Figure 3b). Among the top predictors, Rhinovirus B, Bat Hp betacoronavirus Zhejiang2013, Bat coronavirus BM48-31/BGR/2008, and Human Mastadenovirus C were enriched in Critical cases, possibly reflecting co-infection or virome perturbation during severe disease. In contrast, 16 viral species, including several Streptococcus phages and Cepunavirus Cp7, were enriched in post-recovery individuals, suggesting restoration of commensal virome components during recovery (Figure 3c). This approach not only maximizes interpretability and reduces overfitting risks but also allows for hypothesis generation regarding virome alterations during critical illness and recovery. Future studies with larger cohorts can build upon these findings to validate and expand on the virome’s role in COVID-19 severity and resolution. These results demonstrate that distinct viral taxa not only reflect SARS-CoV-2 infection status but also serve as potential biomarkers for disease severity and recovery trajectories.

### 3.5 Longitudinal paired analysis reveals streptococcus phage enrichment post COVID-19 recovery across disease severities

To understand phage shifts associated with recovery from COVID-19, a longitudinal paired analysis was performed comparing virome profiles from the same patients during active disease (Non-Severe, Severe, Critical stages) and after clinical recovery. This within-patient design enhances the reliability of identifying true recovery-associated microbial signals by controlling for inter-individual variability. Among the paired samples, a significant post-recovery increase in several Streptococcus-specific phages was observed (Figure 4): Streptococcus phage PH10, SpSL1, and EJ-1 exhibited a statistically significant increase in abundance following recovery from the non-severe disease phase (*p* < 0.05 or *p* < 0.01). Additional phages including Streptococcus phages (YMC 2011, PH10, and Cepunavirus Cp1) showed consistent post-recovery uptrends in multiple patients, albeit without reaching statistical significance in all cases. Importantly, in patients recovering from severe and critical COVID-19, phages such as Streptococcus phage PH15, SpSL1, PH10, and Cepunavirus Cp1 also demonstrated clear upward trends post-recovery, despite smaller sample sizes limiting statistical power.

**Figure 4:**
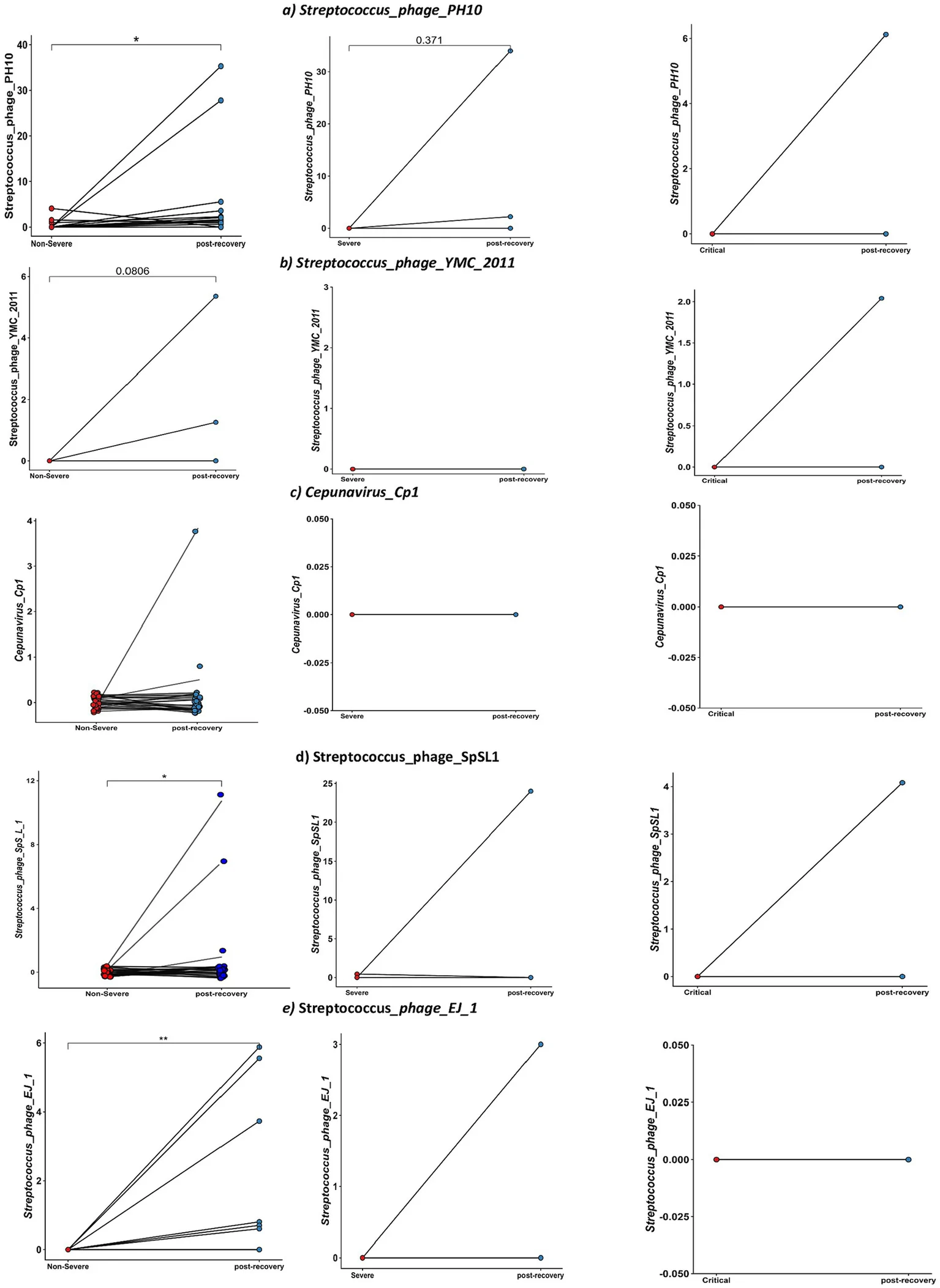
Longitudinal paired analysis of phage abundance in upper respiratory tract samples of COVID-19 patients before and after recovery: Paired line plots show changes in relative abundance of selected Streptococcus phages and Cepunavirus _Cp1 across different disease severity groups (non-severe, Severe, and Critical) and their corresponding post-recovery samples. Each line connects the same individual sampled at the disease phase and after clinical recovery. **(a)** Streptococcus phage PH10, **(b)** Streptococcus phage YMC_2011, **(c)** Cepunavirus Cp1, **(d)** Streptococcus phage SpSL1, and **(e)** Streptococcus phage EJ_1. *p < 0.05, **p < 0.01.

These findings suggest that recovery from COVID-19 is associated with a resurgence of specific bacteriophages, particularly those targeting *Streptococcus* spp., within the same host over time.

### 3.6 Co-occurrence of bacterial taxa in viral-enriched respiratory samples

Although the RVOP kit is designed for the targeted enrichment of viral nucleic acids, our downstream taxonomic classification using Kraken2 revealed a notable proportion of bacterial reads, accounting for approximately 10% of the total classified reads. To further investigate these bacterial profiles, we examined the relative abundance of bacterial taxa across the samples.

As shown in Figure 5, bacterial species such as *Rothia mucilaginosa, Enterococcus faecalis*, and *Prevotella melaninogenica* were among the most abundant across the samples. Interestingly, *R. mucilaginosa* showed a high relative abundance in RT-PCR negative samples (36.1%) and to a lesser extent in post-recovery individuals (3.7%), while *Leptotrichia* sp. oral taxon 212 and *Haemophilus parahaemolyticus* were highly enriched in Critical patients (16.6% and 7.7%, respectively). Several other oral or respiratory commensals, including *Veillonella, Streptococcus, and Prevotella* species, showed group-specific variation, particularly in severe and post-recovery individuals. These findings suggest the presence of non-viral microbial signatures, even in libraries prepared in virus enrichment protocols.

**Figure 5:**
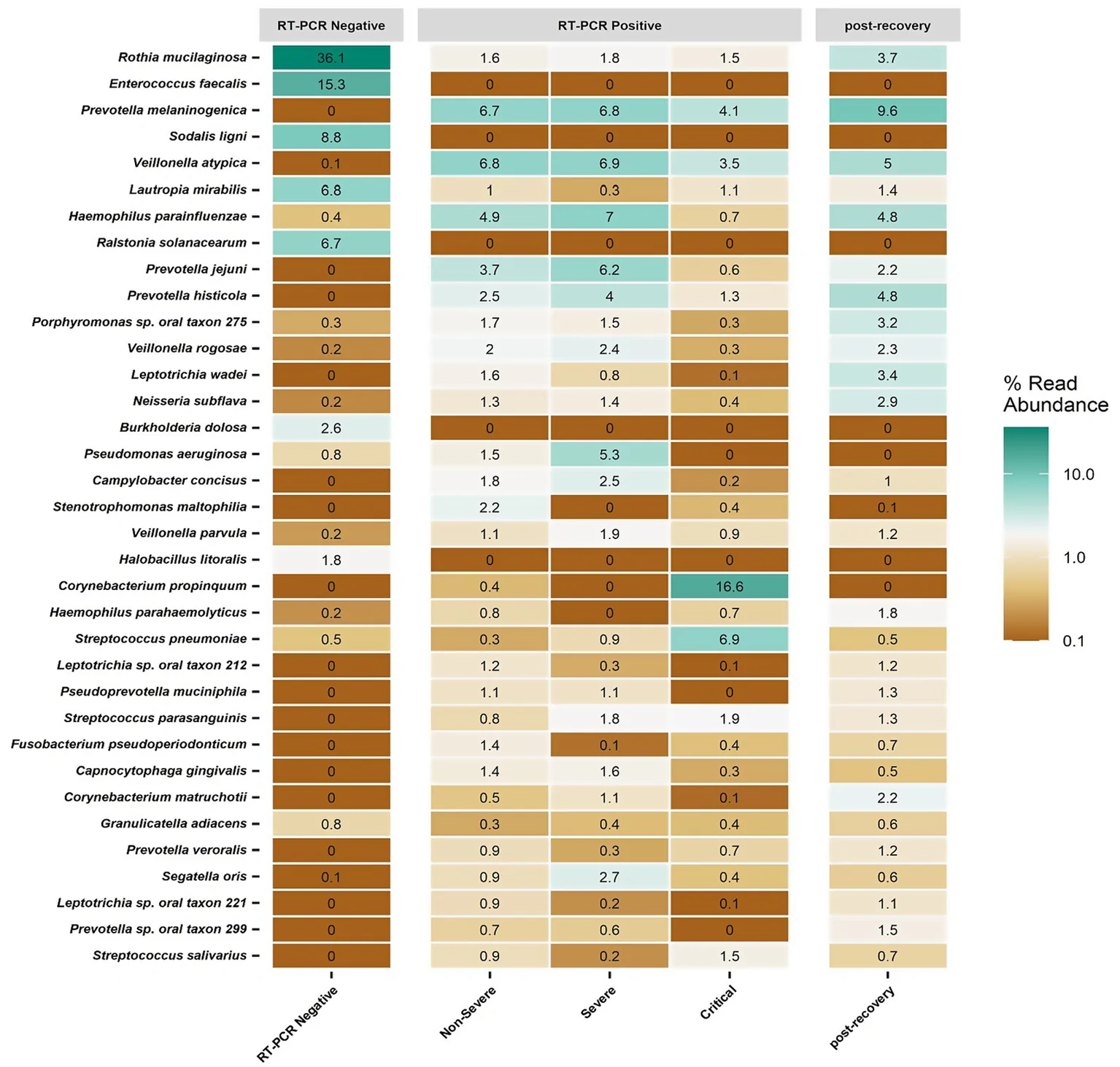
Composition and abundance of upper respiratory tract resident bacteria across COVID-19 severity groups and recovery phase. Heatmap showing the distribution of top 40 bacterial taxa across different clinical categories: RT-PCR Negative, Non-Severe, Severe, Critical, and post-recovery.

## 4 Discussion

The interplay between the virus, the host’s biology, and the resident microbiota plays a central role in determining the severity of COVID-19, influencing both symptom diversity and mortality rates (Devi et al., 2022). Although several studies have been investigated to know the association between upper respiratory tract virome composition and disease severity, the findings remain inconclusive (Ma et al., 2021; Merenstein et al., 2021). To the best of our knowledge, this is the first study to conduct a comprehensive paired analysis of post-recovery samples, providing novel insights into virome dynamics across the course of infection and recovery. Stratification of RT-PCR positive samples was done based on severity.

In this study, we analyzed the composition and diversity of the resident virome in the upper respiratory tract during SARS-CoV-2 infection. The significant increase in both richness (Observed, Chao1) and diversity (Shannon and Simpson) in RT-PCR positive individuals suggests a broader and more balanced virome composition. This may reflect viral proliferation, co-infections or activation of latent viruses in response to SARS-CoV-2 infection and associated immune modulation. The results demonstrate that SARS-CoV-2 infection is associated with a profound shift in the virome composition, as reflected by the significant beta diversity differences between RT-PCR Negative and Positive groups. The greater divergence seen in the post-recovery group suggests a restructured virome, potentially reflecting recovery-associated viral activation, immune modulation, or reintegration of suppressed viral taxa.

Notably, the critical and severe groups also exhibited significantly different viral compositions compared to both the RT-PCR negative individuals and recovered states, potentially due to immune dysfunction, or medical interventions (e.g., antibiotics, antivirals). The modest but significant differences between post-recovery and non-severe/severe groups may reflect ongoing virome rebalancing even after clinical recovery.

Together with alpha diversity data, these results emphasize the dynamic nature of the virome across the infection spectrum, with potential implications for host immunity, recovery trajectory, and long-term health. The exclusive presence of *Escherichia phages* (Zindervirus UAB78 and Joeyvirus) in RT-PCR negative samples may represent a healthy state that is disrupted upon infection. The most abundant virus was found in RT-PCR positive individuals is Human Mastadenovirus C, which is known to cause respiratory tract disease (Chen et al., 2015; Scott et al., 2016).

The paired longitudinal virome profiling in COVID-19 patients provides compelling evidence of within-host virome shifts during the transition from active disease to recovery. Across non-severe, severe, and critical cases, it was consistently observed that resurgence of Streptococcus-infecting phages occurred in the post-recovery state, pointing to a conserved microbial restoration pattern following SARS-CoV-2 clearance. This enrichment could reflect recolonization of native bacteria, immune modulation and phage mediated microbial regulation. Such phage rebound post-COVID-19 has parallels in other respiratory illnesses, where bacteriophage populations dynamically respond to bacterial community restructuring (Cotler et al., 2018; Zuo et al., 2020).

Notably, the persistence of these patterns across non-severe, severe, and critical patients reinforces the idea that specific phage taxa might serve as recovery associated microbial biomarkers, offering new possibilities for virome-based monitoring or microbiome-targeted therapeutics in post-viral care.

### 4.1 Limitation

A key limitation of this study is that bacterial profiles were derived from RVOP viral-enriched libraries rather than dedicated microbiome sequencing (e.g., 16S rRNA or whole-metagenome approaches). Therefore, the bacterial signatures detected should be interpreted cautiously, as the kit is not optimized for comprehensive bacterial profiling. Second, the use of a targeted enrichment panel (Illumina RVOP) may have biased viral detection toward the panel-covered respiratory viruses, potentially underrepresenting novel, low-abundance, or divergent viruses that would have been captured using an untargeted metagenomic approach. The future work should collect and transport samples in separate media so that site specific analysis could provide important insights into difference in virome-bacteriome dynamics between buccal cavity and nasopharyngeal region. Despite these limitations, the findings provide important insights into URT virome alterations associated with COVID-19 severity and recovery.

## 5 Conclusion

COVID-19 is associated with significant but partly reversible restoration of the upper respiratory tract microbiome. Alpha- and beta-diversity analyses demonstrate that overall microbial diversity and community composition altered between controls and cases, and that severe and critical cases showed greater depletion of diversity and the most pronounced compositional shifts, with only partial restoration post-recovery.

Specific eukaryotic viruses and several bacteriophages particularly Streptococcus-infecting phages and phages commonly associated with *E. coli*, emerged as key signatures in healthy controls and post-recovery individuals. Their enrichment suggests a strong association between this virome composition and a healthy microbiome state. Importantly, phages targeting *E. coli* may serve as promising biomarkers of recovery and potential therapeutic targets to mitigate disease severity.

## Data Availability

The datasets presented in this study can be found in online repositories. The names of the repository/repositories and accession number(s) can be found in the article/Supplementary material.
