# Supplementary material for: Upper respiratory virome signatures reflect COVID-19 severity and post-recovery shifts
Source: Front Microbiol. 2026 Oct 12;17:1925299. doi: 10.3389/fmicb.2026.1925299 (PMC13696482; doi:10.3389/fmicb.2026.1925299)
Supplement: Supplementary file 1 [file Data_Sheet_1.PDF]

Supplementary Appendix

Upper Respiratory Virome Variations Track COVID-19 Severity and Post-Recovery Shifts

Mudita Gosain[1#], Manjeet Kaur[2#], Daizee Talukdar[2], Sonali Porey Karmakar[2], Subhash Tanwar[2], Pradipta Jana[2], Jyoti Verma[2], Susmita Bakshi[2], Kajal Kamboj[2], Madhu Pareek[2], Sreevatsan Raghavan[1], Pallavi Kshetrapal[1], Nitya Wadhwa[1], Pramod Kumar Garg[3@], Bhabatosh Das[2@]

1-Pediatric Biology Center, BRIC-Translational Health Science and Technology Institute, NCR Biotech Science Cluster, Faridabad-121001, India
2-Microbial Research Centre, BRIC-Translational Health Science and Technology Institute, Faridabad-121001, India
3 - Department of Gastroenterology, All India Institute of Medical Sciences, New Delhi-110608, India

# *Contributed equally*

Correspondence to:
Bhabatosh Das: *Functional Genomics Laboratory, Centre for Microbial Research, BRIC-Translational Health Science and Technology Institute, NCR Biotech Science Cluster, Faridabad – 121001, Haryana, India.* Phone: +91-129-2876471; *Email: bhabatosh@thsti.res.in*

Pramod Kumar Garg: *Department of Gastroenterology and Human Nutrition, All India Institute of Medical Sciences, New Delhi -110029, India. Email: pgarg@aiims.edu*

Running Title: Respiratory Virome Dynamics in COVID-1

Supplementary Figure 1: Study concept diagram

Supplementary Fig 1a)

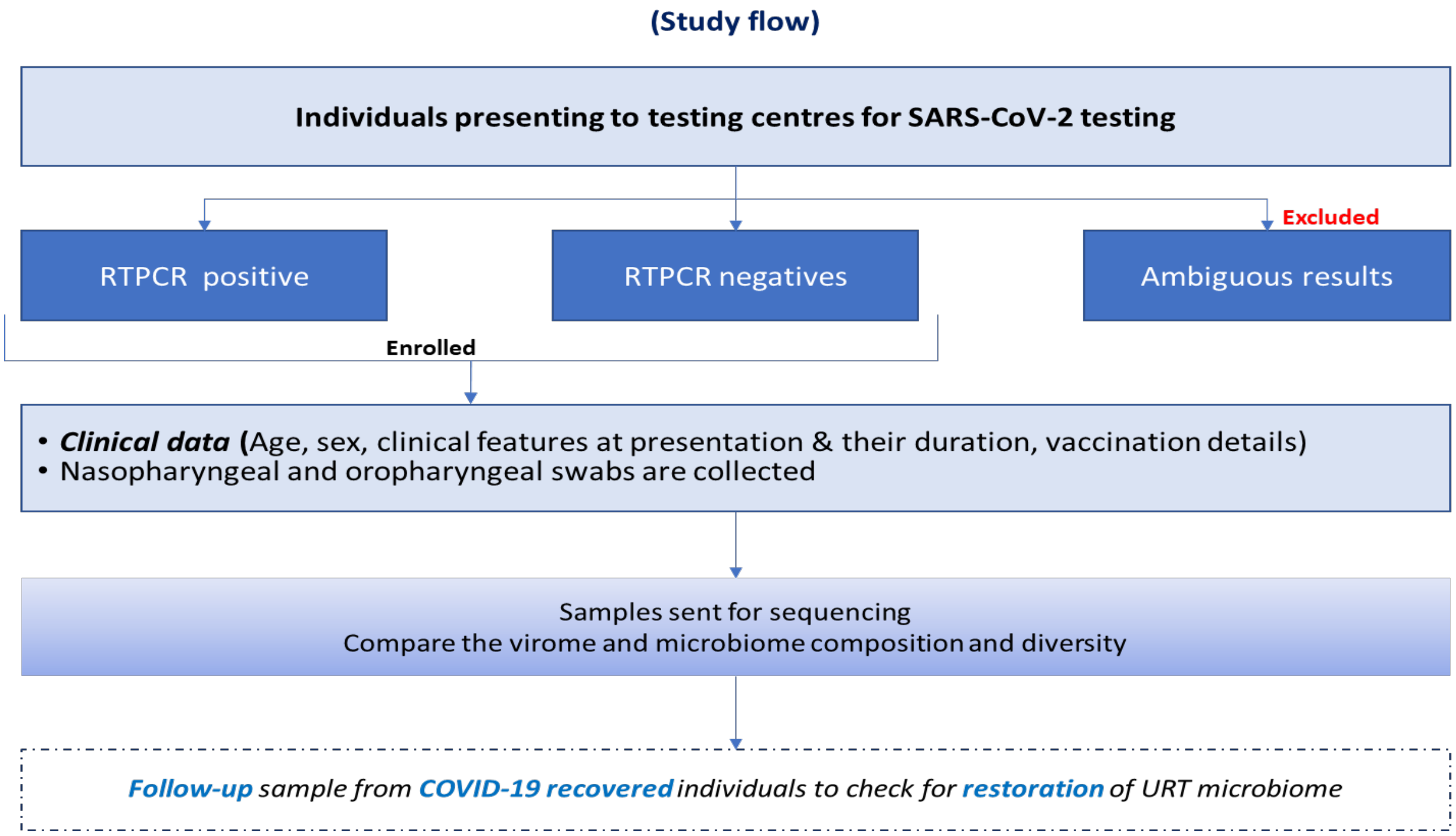

b) Supplementary Figure 1B

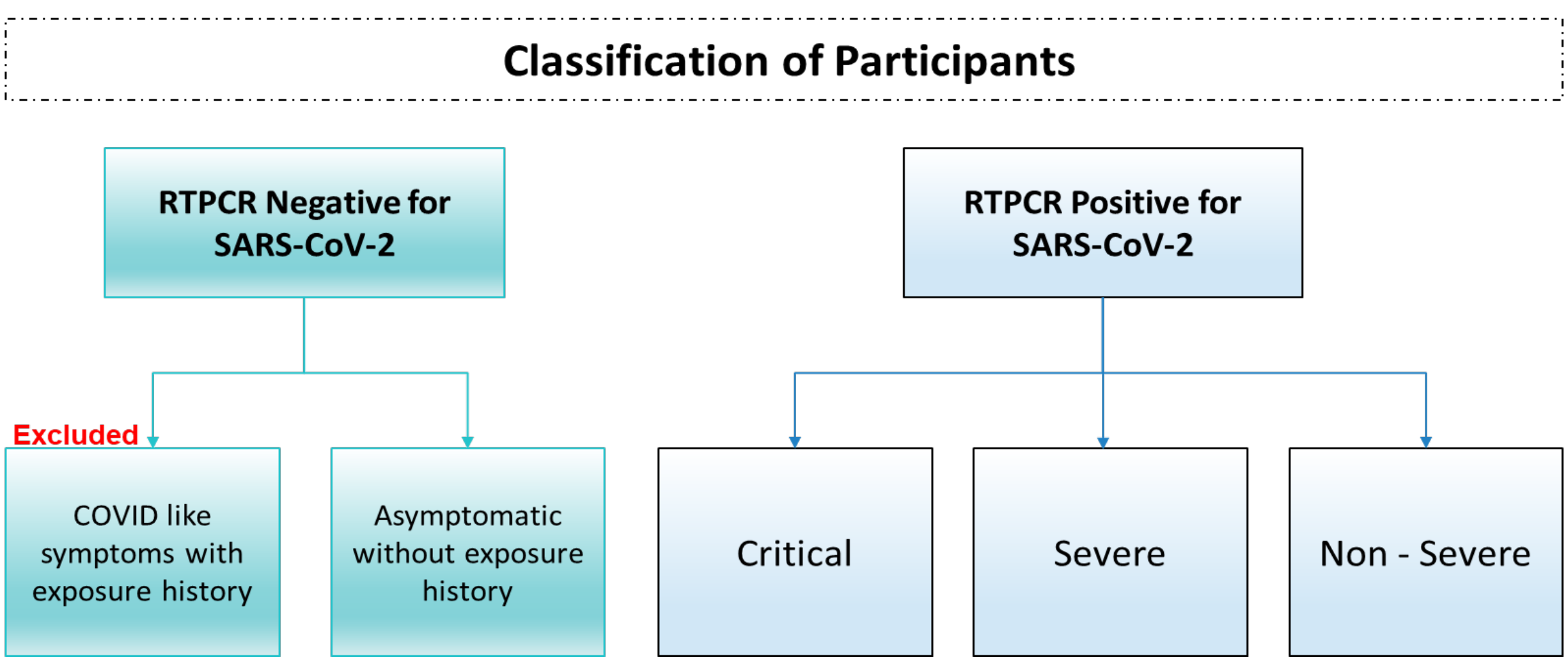

Supplementary Table S1. WHO Clinical Platform for COVID 19: Disease Severity Classification

Category	Definition / Criteria
**Critical**	Death OR admitted to ICU with oxygen (ICU+O) OR admitted to ICU with invasive ventilation (ICU+V) OR ever on high-flow nasal oxygen OR ever on extracorporeal membrane oxygenation (ECMO).
**Severe**	Not classified as critical AND ever on oxygen therapy but not in ICU.
**Non-severe**	Symptomatic or Asymptomatic AND None of the above conditions met.

Note: Definitions of disease severity categories are adapted from the revised WHO Global Clinical Platform for COVID 19 (World Health Organization, 2021)(12)

Supplementary Table S2: Table shows the differential abundance analysis using Lefse showing

Lefse Score

Feature	Enrich_group	ef_lda	padj
s__Jouyvirus_ev207	RT-PCR Negative	5.3222	1.03E-04
s__Delepquintavirus_DLP5	RT-PCR Negative	5.075681	0.006327646
s__Moineauvirus_D1024	RT-PCR Negative	5.037308	0.006327646
s__Pahexavirus_PHL114L00	RT-PCR Negative	4.926839	0.006327646
s__Axomammavirus_PP1	RT-PCR Negative	4.712855	0.006327646
s__Zindervirus_UAB78	RT-PCR Negative	4.508586	0.006327646
s__Human_coronavirus_NL63	RT-PCR Negative	4.417559	0.006327646
s__Munia_coronavirus_HKU13	RT-PCR Negative	4.408222	0.006327646
s__Miniopterus_bat_coronavirus_1	RT-PCR Negative	4.220802	0.006327646
s__Human_mastadenovirus_C	RT-PCR Positive	5.610608	0.002242474
s__Bat_coronavirus_BM48_31_BGR_2008	RT-PCR Positive	5.163254	0.043708042
